# The Effect of Whole-Body Vibration on Proprioception and Motor Function for Individuals with Moderate Parkinson Disease: A Single-Blind Randomized Controlled Trial

**DOI:** 10.1155/2021/9441366

**Published:** 2021-12-17

**Authors:** Kuan-yi Li, Yu-ju Cho, Rou-shayn Chen

**Affiliations:** ^1^Department of Occupational Therapy and Graduate Institute of Behavioral Sciences, Chang Gung University, Taoyuan, Taiwan; ^2^Healthy Aging Research Center, Chang Gung University, Taiwan; ^3^Division of Movement Disorders, Department of Neurology, Chang Gung Memorial Hospital, Linkou Medical Center, Taoyuan, Taiwan

## Abstract

**Introduction:**

Previous studies have shown that whole-body vibration (WBV) may have a potential impact on gait and balance in individuals with Parkinson's disease (PD). However, this body of work has proven inconclusive due to the diverse disease progression and broad age range associated with PD. The effects of WBV on proprioception, a sense frequently affected by PD, has rarely been studied.

**Objective:**

To investigate the short-term effect of WBV on proprioception and motor function for individual with moderate PD.

**Design:**

A single-blind randomized controlled trial. *Setting.* A hospital and a laboratory. *Participants.* 32 participants with moderate PD were recruited and randomly assigned into either the WBV or conventional therapy groups. *Interventions.* For the WBV group, each treatment session included five, one-minute bouts of whole-body vibration paired with one-minute rest (frequency: 6 Hz; amplitude: 3 mm). Each conventional therapy participant received balance and mobility training for 10 minutes. *Main Outcome Measures.* Outcome measures included proprioceptive sensitivity of the upper limb, position sense of the knee joint, Unified Parkinson's disease rating scale : motor section (UPDRS-motor), functional reach test (FRT), and the timed up and go test (TUG).

**Results:**

No statistically significant difference was found between groups. However, both groups showed a significant improvement in motor function after treatment, including UPDRS-motor (*P* = 0.04), less affected side of FRT (*P* = 0.019), and TUG (*P* = 0.006).

**Conclusions:**

Although the effect of WBV was not superior to the conventional therapy, it provided a passive and safe clinical intervention as an alternative treatment, especially for individuals with motor impairment or poor balance function.

## 1. Introduction

For individuals with moderate Parkinson disease (PD), it is necessary to combine both pharmacological and nonpharmacological treatments to improve balance function and functional mobility. In the 19^th^ century, whole-body vibration (WBV) was first introduced by a French neurologist, Jean-Martin Charcot, who later developed a vibration chair to alleviate the symptoms of PD [1]. Since its introduction, WBV has been shown to have a potential impact on sensorimotor function for various populations, including the frail elderly [2, 3], athletes [4–6], healthy adults [7–11], and individuals with stroke [12, 13] and neurodegenerative diseases [14, 15]. WBV is considered to be an easily applied, low person-power, safe clinical intervention, especially for individuals who cannot engage in active movement-focused interventions. As PD is often associated with prominent sensorimotor deficits, WBV has been considered one possible intervention to enhance sensorimotor function [16].

Several previous studies have examined the sensorimotor effect of WBV for individuals with PD [15, 17–27]. Although treatment protocols were not identical among studies, low-frequency and low-amplitude vibrational signals were commonly used for individuals with PD. Frequencies greater than 20 Hz have been reported to generate kinesthetic illusions which may interfere with potential treatment effects [28–30]. In the majority of the reported treatment sessions, 5 bouts of alternating vibration and rest were delivered with each component lasting 1 minute, for a total session duration of 10-minutes [19, 22, 23, 25, 26]. The treatment is delivered to a patient who is placed in a standing possition on the vibration platform with both knees in slight flexion. This postion was proposed to be the ideal position for WBV as it generated the greatest muscle activation compared to other tested positions [3, 31].

Most research has suggested that WBV results in comparable outcomes to that of conventional therapy to improve balance and functional mobility for individuals with PD [19, 20, 23, 25–27]. Two studies reported WBV has superior outcomes as compared to conventional therapy [15, 24]. However, the characteristics of the participants in these studies were heterogeneous (e.g., disease severity and age range) which made it difficult to elucidate whether the treatment effect was confounded by the effects of disease severity or aging. Given these conflicting findings, it is difficult to draw an explicit conclusion about the effect of WBV for individuals with PD.

One notable advantage of WBV is that it requires only a low-level isometric contraction to maintain a slight knee flexion position which has shown benefit for individuals with moderate to severe motor impairments. Previous work has suggested that the sensory stimulus from the vibration is transmitted to the primary endings of the muscle spindle fibers which in turn activates the alpha motor neuron and results in increased muscle contraction and subsequent brain activation [32–36]. WBV could increase proprioceptive input and consequently lead to improvement in motor and balance function [33–35]. Although considerable research has been done to examine the treatment effect of WBV on motor and balance function, few studies have been done to investigate the potential impact of WBV on proprioception to clarify the connection between proprioceptive function and improved motor function. Currently, only one study measured knee joint proprioception to investigate the potential impact of WBV on proprioceptive function for individuals with PD [22]. The study did not find significant changes in proprioceptive performance at the knee joint following WBV; however, the authors cautioned that the complexity of measuring proprioception may allow for possible changes in performance that might not be measurable or detected by their assessment techniquies. Therefore, one major purpose of this study was to examine the effect of WBV on proprioception using different assessment modalities including a passive motion apparatus and a movement reproduction tasks. In addition, we examined the upper and lower extremities for individuals with moderate PD to fully explore possible performance changes at different limbs as well as more and less affected sides. The hypotheses of this study are as follows: (1) that the effect of WBV on proprioception is significantly different from the conventional therapy group and (2) the effect of WBV is equal to or greater than the effect of conventional therapy on measures of motor and balance function.

## 2. Methods

### 2.1. Participants

We conducted a single-blind, randomized controlled trial between December 2017 and June 2018 in a lab setting. An independent rater, blind to the group allocation, evaluated the sensorimotor function before and after treatment. The effect size for sample size calculation was based on a previous study done by Gassner (2006) who reported the difference of UPDRS-motor score between two groups for people with PD. The effect size was 0.5; *α* = 0.05, *β* = 0.8 resulted in a sample of 15 people per group. The inclusion criteria for all participants were as follows: (1) age range between 50 and 65 years, (2) diagnosed as having an idiopathic PD, (3) moderate stage progression based on Hoehn and Yahr stage classification and UPDRS scale [37], (4) no cognitive impairment (Mini-Mental State Examination (MMSE) score ≥ 24) [38], and (5) neurological examination clear of any signs or symptoms of peripheral nerve disorders, such as peripheral neuropathy. Exclusion criteria included (1) other neurological disorders (e.g., stroke) which might interfere with the ability to detect arm position and motion; (2) any medical history of injury to the extremities that may affect proprioceptive sensitivity, e.g., shoulder dislocation or joint replacement; (3) inability to follow instructions and focus on the experiment for 30 minutes; and (4) tremor-dominant presentation. Tremor-dominant patients were not included because the involuntary movement might interfere with the ability to detect arm position and motion. All participants were provided the consent form and provided written voluntary consent to participate in the study. The study was approved by the institutional review board of Chang Gung Memorial Hospital (201702010B0). Clinical trial registration number is 201702010B0C603 (ClinicalTrials.gov).

All participants were tested while taking their routine antiparkinsonian medications (ON phase). Daily doses of medication were standardized using the following formula: 100 mg standard levodopa is equal to 125 mg sustained release levodopa, 1.5 mg pramipexole, 6 mg ropinirole, 10 mg bromocriptine, or 1 mg pergolide [39].

### 2.2. Procedure

All participants completed two total study visits. During the initial visit and prior to beginning the testing, each participant completed the consent process and underwent an initial screening, including demographic information and history of neurological disease. Those who met the inclusion criteria completed the UPDRS-motor subsection, Hoehn and Yahr stage, and MMSE assessments. Eligible participants were then randomly assigned into the experimental group or conventional therapy group to using the computerized block randomization. In order to maximize treatment adherence, each participant received two treatment sessions at a 2-month interval which corresponded with their regularly scheduled visits to the outpatient clinic to get their levodopa prescriptions. As most participants traveled a substantial distance, coordination of their appointments best served the participants and increased the likelihood of compliance with the study protocol.

All assessments were performed before and immediately after each treatment session. That is, some participants' proprioceptive sensitivity was evaluated only before and after the first treatment session. When they returned two months later, the other assessments (knee joint proprioception and motor function assessment) were performed before and after the second treatment session. The order of proprioceptive sensitivity and the other assessments' delivery was random.

### 2.3. Assessment

#### 2.3.1. Proprioceptive Sensitivity in the More Affected Arm

A passive motion apparatus was used to measure passive motion sense for the more affected arm. An apparatus with similar specifications has previously been used in studies of individuals with Parkinson disease [40], typically developing children [41] as well as children with developmental coordination disorders [42]. The device consisted of a rectangular metal splint (60 × 9 cm) supported by a metal drive shaft. The torque engine powering the apparatus is capable of generating angular velocities as low as 0.02°/s and as fast as 300°/s.

The height of the chair and apparatus were adjusted according to each participant's seated height. Each participant's forearm was placed on the rectangular splint in a starting position of slight shoulder abduction and 90° of elbow flexion. A hand-held goniometer was used to ensure the consistency of the starting position. Participants wore goggles and headphones with pink noise to occlude visual and auditory cues during testing and ensure that proprioceptive cues were used to make perceptual judgment. At the beginning of each trial, a tactile cue on the shoulder with the verbal command “concentrate now” was given as a starting signal.

Each passive motion sense trial consisted of two angular velocities with a standard velocity of 1.5°/s and a comparison velocity that ranged between 1.58°/s and 2.63°/s with a step of 0.15°/s. The interstimulus interval (ISI) between the standard and the comparison velocity was 500 ms. At the end of each trial, participants had to indicate which angular velocity was faster, the first or the second. A standard forced-choice paradigm was used; therefore, participants could not respond “I don't know” or “they were the same.”

The order of standard stimulus and comparison stimulus was presented randomly to control for any potential order effect. Each trial could be repeated once if the participant was distracted and unable to make a judgment during the data collection. The experimenter recorded each participant's verbal responses.

**Figure 1:**
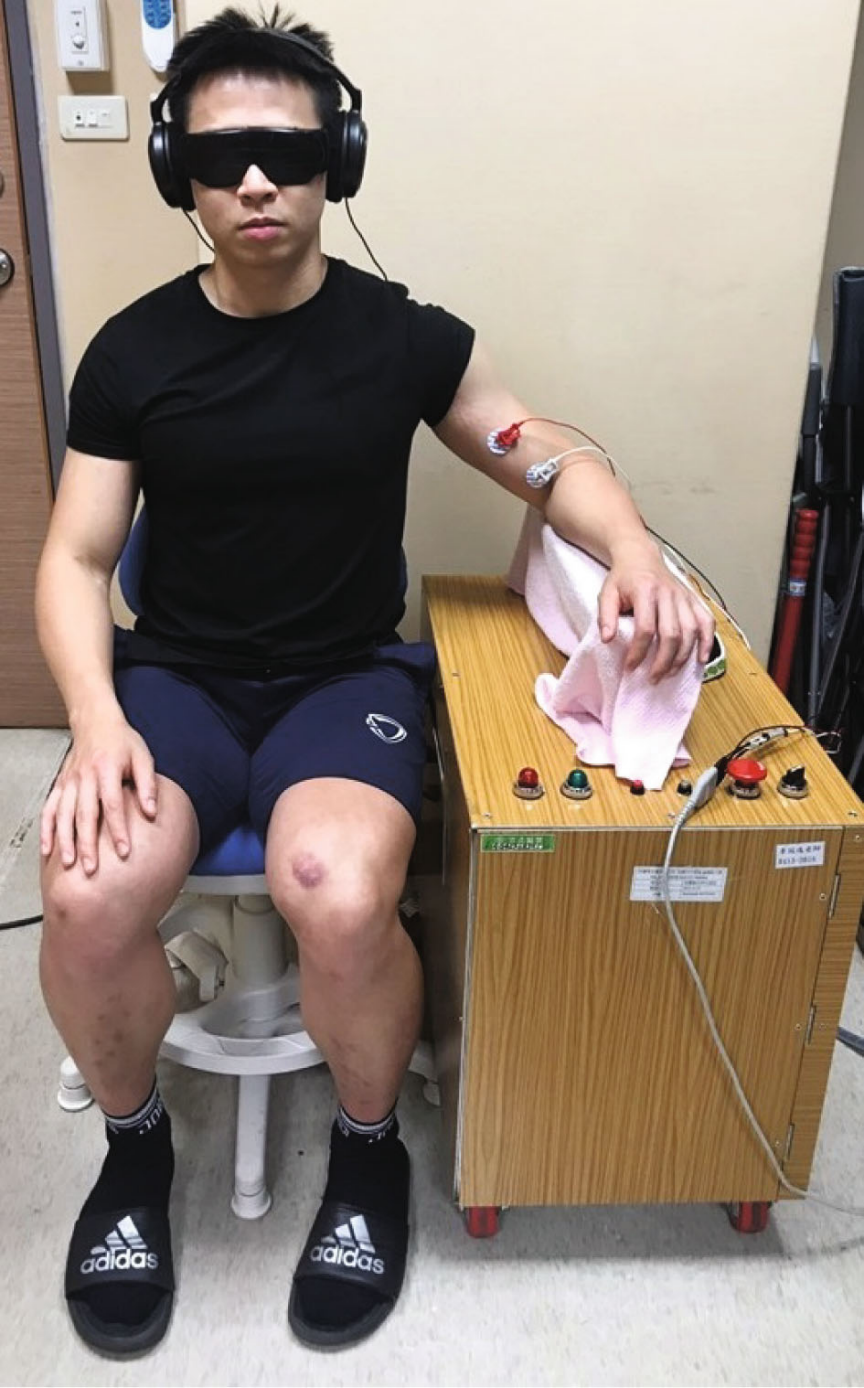
Experimental setup for the passive motion sense assessment.

Throughout the experiment, myoelectric activities of the biceps was monitored online by standard surface electromyography (EMG) with sampling rate at 1000 Hz to ensure that participants did not generate any movement during the test. Any trial with exhibited EMG activities was excluded and then repeated afterward. The more affected arm was tested for all participants. Before the data collection, three practice trials were administered to confirm that participant understood the experimental procedure. A total of 72 trials were administered. The experimental setup is shown in Figure 1.

#### 2.3.2. Knee Joint Proprioception Assessment

Participants sat comfortably with their knee flexed to 90° as the beginning position. The individual was told to remain still, and their leg would be passively moved by an experimenter. The experimenter moved the testing leg to the target position for 15 seconds and returned to the starting position. Participants were then asked to actively move the same leg to the remembered target position. The experimenter measured and recorded the difference between the target position and the matched position with a digital hand-held goniometer. Three target positions were tested, and they were knee flexion at 15°, 30°, and 70°. Each position was tested three times, and both legs were tested [43].

#### 2.3.3. Unified Parkinson Disease Rating Scale: Motor Section (UPDRS-motor)

Unified Parkinson Disease Rating Scale is the most common and widely used assessment to evaluate disease progression for individuals with PD [44]. There are 4 subtests: mental status, mood, and behaviors; activities of daily living (ADL); motor; and complications of treatment. Only the motor subtest was assessed for this study. Previous studies have established good internal consistency [45], rater reliability, and test-retest reliability [46].

#### 2.3.4. Functional Reach Test (FRT)

The functional reach test was used to evaluate balance function. Participants were required to stand against the wall and flex the testing arm to 90° with a fist as the starting position. Then, they were asked to push their fists forward as far as they could without moving either foot or falling. The experimenter measured the distance between the start and final positions at the third metacarpal joint. Each arm was assessed three times, and the average of the last two trials was taken as the functional reach distance [47].

#### 2.3.5. Timed Up and Go Test (TUG)

The TUG assesses an individual's functional ambulation. Each trial began with the participant seated in a chair. Following a cue from the experimenter, the participant stood up and walked at their comfortable and safe speed for 3 meters to a line marked on the floor, turned around, walked back to the chair, and sat down again [48]. The test was repeated three times, and each trial was timed and recorded. The mean of the three trials was used as the TUG time [48]. A previous study suggested that the TUG time could be a quantitative indicator of function ambulation for individuals with PD [49].

### 2.4. Treatment Protocols

#### 2.4.1. Whole-Body Vibration Group

No standardized clinical protocol for the delivery of WBV has been established; therefore, it is difficult to compare the treatment effects between studies. Recently, the reporting guidelines for WBV studies in humans, animals, and cell cultures have been discussed and published [50, 51]. The reporting guidelines were established for designing future WBV studies and enhancing the quality of WBV publications. To investigate the potential effect of vibration stimuli on proprioception in PD, the immediate effect of WBV was examined in this study. The treatment protocol used was based on previous research reporting significant improvement in postural control for PD [18, 19, 22, 23, 25, 26]. At the beginning of the treatment, verbal instructions explaining the procedure of the WBV sessions were given to the participants. The vibration stimuli were delivered via a plate at the center of a custommade vibration device (length: 72 cm; wide: 70 cm; and height: 119 cm) (Tokuyo, TS-808AA, Taiwan) on the ground, with the plate attached to the center (length: 51.5 cm; wide: 36.5 cm). The vibration device produced a sinusoidal vibration with a primary vertical component and a much smaller horizontal component. The direction of the acceleration was time-invariant. Participants were instructed to remain static, in a standing position, with their shoes on the whole body vibration platform. Their heads and eyes faced forward, with a slight knee flexion, feet were shoulder-width apart, and both hands on the rails for safety. The vibration frequency and amplitude settings were 6 Hz and 3 mm, respectively, which were the most commonly used vibrational settings in the literature [18, 19, 21–23, 26]. Under this setting, the feet were predominantly subjected to the vibration stimuli. Each treatment session consisted of 5 bouts of vibration for 1 minute and rest for 1 minute. During the WBV treatment sessions, the experimenter stood next to the platform to supervise and guard the safety of the participants. Both the WBV and conventional therapy groups received treatment on weekday mornings at the hospital.

#### 2.4.2. Conventional Therapy Group

Participants received 10 minutes of conventional therapy for postural control and functional ambulation, including sit-to-stand exercise, practice of functional activities, and weight-bearing activities. A certified occupational therapist individualized the grade of the activity for each participant during training.

#### 2.4.3. Data Analysis and Statistics

Proprioceptive sensitivity data from the more affected arm were analyzed using MATLAB. The percentage of correct response for each stimulus intensity was tallied, and a psychometric function was calculated for each individual. The just noticeable difference threshold (JNDT) for passive limb motion sense was defined as the perceived intensity with 75% correct response.

IBM SPSS Statistics for Windows, version 22.0, was used for statistical analysis. A two-way repeated measure analysis of variance was used to compare the within-group (time effect) and between-group (the WBV vs. conventional therapy) differences. A post hoc analysis with Bonferroni correction was used if necessary. A Pearson correlation coefficient was used to examine the relationship between proprioception and motor performance. The level of significance was set at *α* = 0.05.

## 3. Results

**Figure 2:**
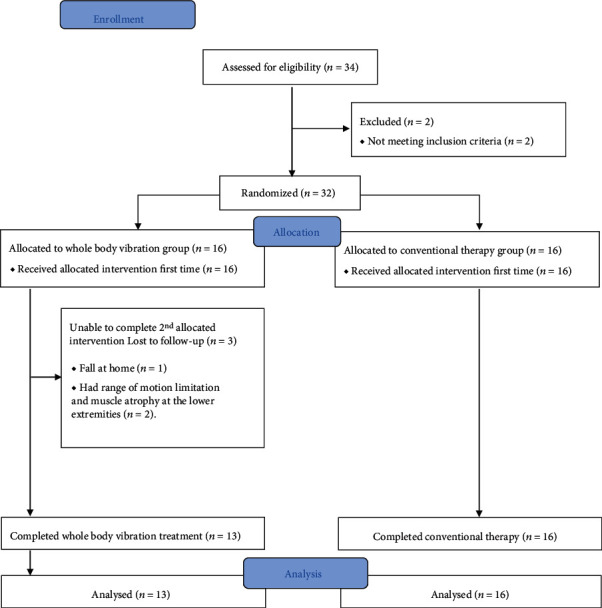
CONSORT flow diagram.

Thirty-two individuals with moderate PD were recruited from an outpatient neurology clinic, and none of them received WBV treatment before. Three participants assigned to the WBV group were unable to complete the study: one participant fell during normal activity in the community after the first treatment, one participant had limited range of motion at the knee joint after the first treatment, and the third individual showed muscle atrophy at the right leg before the treatment. Therefore, data from 29 participants were analyzed (mean age: 60.55 ± 3.51; range, 53-65y; 18 male and 11 female; 27 right handed; 17 right side-onset and 12 left side-onset (Figure 2). No significant difference was present between two groups at baseline including demographic characteristics and outcome measures. Detailed demographic data are provided in Table 1.

Significant main effects of time were found for UPDRS-motor (*F*_1,27_ = 4.662, *P* = 0.04), FRT less affected arm (*F*_1,27_ = 6.174, *P* = 0.019), and TUG (*F*_1,27_ = 8.715, *P* = 0.006). No significant group×time interaction effect and no significant main effect of group were found. That is, the whole sample of 29 individuals, including those assigned to WBV and conventional therapy, improved motor function, but no significant difference between conventional therapy and WBV was observed. There was no significant relationship between proprioception and motor performance (all *P* > 0.05). However, the change of UPDRS-motor was significantly correlated with the change of TUG (*r* = 0.73; *P* < 0.001), FRT of the more affected arm (*r* = −0.43; *P* = 0.02), and FRT of the less affected arm (*r* = −0.38; *P* = 0.04). Detailed results for each outcome measures are presented in Table 2.

## 4. Discussion

Utilizing previously established methods to measure proprioception for the upper and lower extremities, this study was designed to explore the effect of WBV on proprioception for individuals with PD, a pathology known to affect proprioception and proprioceptive sensitivity. This is the first randomized controlled trial to examine the short-term effect of WBV on proprioception for both the upper and lower extremities in individuals with Parkinson disease. The results suggested that the WBV treatment did not significantly improve proprioceptive function for individuals with moderate PD. However, the WBV showed a similar effect to conventional therapy in improving motor function and balance which was consistent with previous findings [18, 20–22].

Previous research postulated that the observed effect of WBV could be due to increased activation of both sensory and motor neurons [7]. Findings from an animal study provided further evidence which indicated that WBV could significantly increase the responsiveness of choline acetyltransferase-immunoreactivity in the somatosensory cortex and basolateral amygdala [52]. Consequently, the increased release of acetylcholine would lead to the fast transmission of signals from the somatosensory cortex and basolateral amygdala. Although several studies proposed that the increased sensory inputs cause the observed improvement of motor function based on the findings mentioned above, none of studies examined the effect of WBV on sensory function except one [22]. Therefore, we investigated whether WBV could improve proprioception and motor function in both the upper and lower extremities.

One of our research hypotheses was that WBV would improve proprioception and motor function in moderate PD; however, current findings did not show significant improvement of proprioception in either the upper or lower extremities. Although different methods were used to measure proprioception, the current results were consistent with a previous study which showed no evidence finding of improved proprioception after WBV treatment [22]. However, both the current study and the previous research measured proprioceptive sensitivity or proprioceptive function instead of the activation of type Ia and type II sensory neurons. This study used a passive motion apparatus and position matching task to measure proprioception in the upper and lower extremities, respectively. For both of these tasks, the outcome measures are dependent upon judgements of the participants rather than actual physiological measurements. It is possible that the gain of increased activation of type Ia and type II sensory neurons maybe not large enough to enhance the proprioceptive sensitivity but is none the less present. Additionally, our testing paradigm required the participant to attend to the psychophysical task and focus on relatively small perturbations. Although participants were required to take several breaks during the test, we cannot completely exclude the potential impact of inattention on sensory performance. Finally, this study purposely measured proprioception in isolation from other sensory stimuli; therefore, we cannot fully exclude the potential impact of WBV on sensory function in moderate PD.

Results from the motor function and balance assessments did not show a significant group by the time interaction effect which indicated that the effect of WBV was not superior to the conventional therapy paradigm. Both the experimental and conventional therapy groups showed significant improvements in UPDRS-motor, FRT of the less affected side, and TUG following the intervention, which is consistent with previous findings [18, 20, 21, 23]. Both groups were well matched at baseline and group assignment followed standard randomization techniques. Experimental design and protocols were similar with previous research [19, 22, 23, 25, 26]. A low-frequency and low-amplitude vibration stimuli was used for the WBV paradigm as was the convention in the majority of the previous literature. For vibrational signals with low frequency, a previous study reported that 3, 6, and 9 Hz WBV did not show a different treatment effect for PD [19]. Therefore, we concluded that the WBV paradigm used in this study had a potential impact on motor and balance function for individuals with moderate PD and the effect was similar to the effect observed following conventional therapy.

Different from previous studies, participants in this study were very closely matched on disease severity and age. All participants were scored as having moderate PD with Hoehn and Yahr stage scores between 2 and 2.5. Participant's ages ranged from 50 to 65 years. FRT pretest scores were within the range of normal as compared to age-matched healthy elderly [47]. This high degree of function may partially explain why no significant difference was found between WBV and conventional therapy as these individuals appear to be at a relatively high functional level. However, the average time taken for the TUG test was within the range of 80-89-year-old healthy elderly individuals [53]. Taken together, participants in the study showed sound dynamic balance and very poor mobility.

## 5. Conclusion

Current findings suggested that WBV did not result in between-group significant short-term benefit to proprioception and motor function for individuals with moderate PD. However, both WBV and conventional therapy groups showed a significant difference in UPDRS-motor, FRT of the less affected arm, and TUG after treatment. In line with previous studies, WBV resulted in outcomes similar to those of conventional therapy in improving motor and balance function. Taken together, WBV could be considered a possible passive motion alternative treatment for individuals with moderate PD.

## 6. Limitations

There were several limitations to our study. First, the sample size was small. However, our participants were more homogeneous than those included in previous studies, the results were representative for the short-term effect of WBV for those with moderate PD (H&Y stage between 2 and 2.5). Second, the techniques used to measure proprioception were complex psychophysical methods and the results might interfere with the motor symptoms of PD. Future studies should consider the potential impact of PD symptoms on measuring proprioceptive sensitivity and apply different method to measure proprioception with increased sensitivity and with greater regard for physiological function. Finally, this study was limited to the short-term effect of WBV for individuals with moderate PD and the results are not generalizable beyond this scope; therefore, long-term effect of WBV remains unknown.

## Figures and Tables

**Table 1:** Demographic characteristics of the participants.

	WBV group (*N* = 13)	CT group (*N* = 16)	*P* value
Age (mean ± SD)	61.15 ± 3.72	60.06 ± 3.38	0.42
Sex (male/female)	11/2	7/9	
The more affected arm (right/left)	13/0	14/2	
MMSE (mean ± SD)	28.92 ± 1.12	28.56 ± 1.41	0.46
Hoehn & Yahr stage (mean ± SD)	2.23 ± 0.39	2.34 ± 0.35	0.42
Levodopa equivalence dose (mg)	226.93 ± 171.53	156.29 ± 137.69	0.23

Abbreviation: WBV: whole body vibration; CT: conventional therapy; MMSE: Mini Mental State Examination; UPDRS: Unified Parkinson Disease Rating Scale.

**Table 2:** Means and standard deviations for the outcome measures before and after treatment.

	WBV group (*N* = 13)	CT group (*N* = 16)	*P* value		
			Interaction effect (group by time)	Main effect of time	Main effect of group
Proprioceptive sensitivity—more affected arm (°/s)			0.31	0.70	0.10
Pretest	0.59 ± 0.30	0.52 ± 0.30			
Posttest	0.71 ± 0.37	0.47 ± 0.30			
Knee joint proprioception (°)					
More affected side					
70°			0.11	0.89	0.41
Pretest	3.26 ± 2.24	4.81 ± 3.58			
Posttest	3.97 ± 2.41	4.21 ± 3.47			
30°			0.71	0.84	0.92
Pretest	3.69 ± 2.06	3.79 ± 2.39			
Posttest	3.77 ± 2.35	3.54 ± 1.70			
15°			0.98	0.98	0.04
Pretest	4.31 ± 2.35	2.81 ± 1.68			
Posttest	4.31 ± 2.92	2.79 ± 1.37			
Less affected side					
70°			0.67	0.95	0.90
Pretest	3.95 ± 2.05	3.77 ± 2.08			
Posttest	3.64 ± 1.95	4.00 ± 3.35			
30°			0.90	0.83	0.51
Pretest	3.69 ± 2.38	3.27 ± 1.66			
Posttest	3.54 ± 1.93	3.23 ± 1.66			
15°			0.45	0.67	0.36
Pretest	3.92 ± 2.91	3.00 ± 1.31			
Posttest	3.41 ± 2.11	3.15 ± 1.86			
UPDRS-motor			0.53	0.04^∗^	0.004^∗^
Pretest	22.77 ± 10.73	23.75 ± 9.80			
Posttest	21.62 ± 9.22	23.13 ± 9.74			
FRT (cm)					
More affected side			0.71	0.88	0.19
Pretest	31.31 ± 8.05	35.45 ± 7.41			
Posttest	31.53 ± 9.55	34.92 ± 7.00			
Less affected side			0.79	0.02^∗^	0.3
Pretest	30.09 ± 10.05	32.92 ± 8.43			
Posttest	32.20 ± 9.10	35.54 ± 6.17			
TUG (s)			0.41	0.006^∗^	0.7
Pretest	11.70 ± 5.36	10.80 ± 3.02			
Posttest	10.14 ± 4.80	9.93 ± 2.40			

## Data Availability

The datasets generated and analyzed during the current study are available from the first author on reasonable request (Kuan-yi Li; kyli@mail.cgu.edu.tw).
